# An Automated Micro Solid-Phase Extraction (μSPE) Liquid Chromatography-Mass Spectrometry Method for Cyclophosphamide and Iphosphamide: Biological Monitoring in Antineoplastic Drug (AD) Occupational Exposure

**DOI:** 10.3390/molecules29030638

**Published:** 2024-01-30

**Authors:** Stefano Dugheri, Donato Squillaci, Valentina Saccomando, Giorgio Marrubini, Elisabetta Bucaletti, Ilaria Rapi, Niccolò Fanfani, Giovanni Cappelli, Nicola Mucci

**Affiliations:** 1 Department of Experimental and Clinical Medicine, University of Florence, 50134 Florence, Italy; donato.squillaci@unifi.it (D.S.); valentina.saccomando@edu.unifi.it (V.S.); elisabetta.bucaletti@unifi.it (E.B.); ilaria.rapi@edu.unifi.it (I.R.); niccolo.fanfani@unifi.it (N.F.); giovanni.cappelli@unifi.it (G.C.); nicola.mucci@unifi.it (N.M.); 2 Department of Drug Sciences, University of Pavia, Via Taramelli 12, 27100 Pavia, Italy; giorgio.marrubini@unipv.it

**Keywords:** antineoplastic drugs, biological monitoring, ultrahigh-performance liquid chromatography, tandem mass spectrometry, µSPEed, micro solid phase extraction

## Abstract

Despite the considerable steps taken in the last decade in the context of antineoplastic drug (AD) handling procedures, their mutagenic effect still poses a threat to healthcare personnel actively involved in compounding and administration units. Biological monitoring procedures usually require large volumes of sample and extraction solvents, or do not provide adequate sensitivity. It is here proposed a fast and automated method to evaluate the urinary levels of cyclophosphamide and iphosphamide, composed of a miniaturized solid phase extraction (µSPE) followed by ultrahigh-performance liquid chromatography-tandem mass spectrometry (UHPLC-MS/MS) analysis. The extraction procedure, developed through design of experiments (DoE) on the ePrep One Workstation, required a total time of 9.5 min per sample, with recoveries of 77–79% and a solvent consumption lower than 1.5 mL per 1 mL of urine sample. Thanks to the UHPLC-MS/MS method, the limits of quantification (LOQ) obtained were lower than 10 pg/mL. The analytical procedure was successfully applied to 23 urine samples from compounding wards of four Italian hospitals, which resulted in contaminations between 27 and 182 pg/mL.

## 1. Introduction

Antineoplastic drugs (ADs) are a heterogeneous and widely used class of compounds with a rapidly growing market [1]. ADs are mainly used as chemotherapy in the treatment of neoplastic diseases, but they also play an important role in haematology and rheumatology and are used to treat non-cancer diseases such as multiple sclerosis, psoriasis, and systemic lupus erythematosus. Their various mechanisms of action usually involve inflicting genetic damage to cancerous cells without specific targeting, which inevitably leads to important side effects at the expense of healthy cells, both of treated patients and healthcare personnel. Despite the considerable steps taken in the last decade in the context of safety regulations relating to their handling [2,3], Ads’ mutagenic effect still presents a tangible risk concerning occupational exposure, due to the possibility of dermal absorption from contaminated surfaces, which is the most common route of exposure in the hospital environment. The European Parliament and Council published the third revision of the Carcinogen and Mutagens Directive (CMD) 2004/37/EC [4] recognizing and prioritizing for the first time this important issue for healthcare workers and patients exposed to hazardous medicinal products (HMPs). In 2020, the European Commission conducted a study and consultation to further amend the CMD [5,6,7], which resulted in the last revision (Directive 2022/431/EU) [8] in March 2022 being adopted by national laws in all EU member states by 5 April 2024. Both Directive 2022/431/EU and the resulting guidelines, “Guidance for the safe management of hazardous medicinal products at work” [9], published in 2023, encourage the development of monitoring methods and biological surveillance for exposed health professionals. While surface contamination sampling remains an important tool to detect incorrect working procedures and increase the awareness of hospital workers [10,11,12,13,14], biological monitoring is still the only way to truly assess occupational exposure and identify possible pathological correlations. Nowadays, ADs can still often be found in the biological fluids of exposed healthcare personnel [15]. Numerous analytical methods can be encountered in the literature for the extraction of ADs in urine samples, mainly based on solid-phase extraction (SPE) or liquid-liquid extraction (LLE), but they usually require large volumes of samples and extraction solvents, or do not provide adequate sensitivity [16,17,18,19,20,21] due to the progressive decrease in encountered contaminations [22]. Solvents’ consumption shown in previously cited studies can vary from 5 to 45 mL and, in many cases, by the use of not environmentally friendly substances such as dichloromethane and n-hexane. Thus, the development of growingly sensitive, green and fast methods of analysis via the means of innovative technologies is the only way to keep up with the decreasing levels of contamination which can be encountered after the latest safety implements, following the “as low as reasonably achievable” (ALARA) principle.

Many miniaturized solid-phase extraction techniques (µSPE) are being developed and applied to sample preparation in numerous fields of application [23,24]. This sample treatment technique aims to improve selectivity and sensitivity through sample clean-up and pre-concentration. It generally involves miniaturized cartridge-type devices that contain packed solid particles of porous chromatographic material. The mechanism of action of SPE consists in the interaction between the solid sorbent phase and the analytes contained inside a liquid sample solution that is percolated through the SPE bed. The analytes bonded to the sorbent are then recovered thanks to the affinity of a small amount of solvent. In this context, the µSPEed cartridges, patented by ePrep Pty Ltd. (Melbourne, Australia) (United States Patent US 2015/0352543 A1), represent a valid innovation. The cartridge is comparable to a short HPLC column: it presents a 3 µm sorbent particle size, offering a higher surface area (instead of the 50 µm diameter particles traditionally used in SPE) and thus a more efficient separation, along with a pressure-driven one-way check valve that allows an ultra-low dead volume connection. The valve design allows the sample to be drawn into a syringe avoiding transit through the sorbent bed and then passed through the stationary phase by simply pulling and pushing the plunger. The cartridges are reusable (depending on the sample matrix and operating procedure) after adequate rinsing and can be coupled with the digiVOL^®^ Digital Syringe Driver or the ePrep ONE workstation, both marketed by ePrep Pty Ltd. (Melbourne, Australia), to automate the procedure. 

The aim of this work was to develop a fast and automatable sample preparation method which could be coupled to ultrahigh-performance liquid chromatography-tandem mass spectrometry (UHPLC-MS/MS) analysis and offer competitive results in comparison to the existing methods, whose detection limits generally vary between 5 pg/mL and 30 ng/mL, with lower environmental impact. The selected target molecules, iphosphamide and cyclophosphamide, are two widely prescribed DNA-alkylating agents which are strongly excreted unchanged in urine and thus commonly employed as markers of exposure to ADs. The approach of Design of Experiments (DoE) was chosen to optimize the multiple parameters involved in the micro-extraction setup, allowing a reduction in the number of experiments needed for the optimization of the method and thus the amount of solvents and resources utilized. Moreover, to evaluate the environmental friendliness of the analytical method proposed, the Green Analytical Procedure Index (GAPI) tool was applied [25].

## 2. Results and Discussion

For the time being, biological monitoring for AD exposure presents many issues, including the need to detect low urinary levels and use different extraction methods for each class of analytes, which entails complex and time-consuming procedures. Furthermore, the complexity of the dermal route of exposure makes the correlation between surface contamination and urinary AD levels extremely difficult.

The drugs in the study, cyclophosphamide and iphosphamide, represent a convenient starting point for new method development, thanks to their feature of being found unchanged in urine samples from exposed personnel and their strong response in electrospray source (ESI) mass spectrometry. As a matter of fact, for most Ads, only the metabolized drug can be found in urine in detectable concentrations, but since these metabolites are often commercially unavailable, a correct quantification is strictly tied to expensive and specific synthetic procedures.

The developed method offers a fast and sensitive alternative to the currently used urine extraction procedures, along with comparable performances. The DoE approach is in different ways a key factor in the optimization of the mobile phase, minimizing the number of experiments and giving an intuitive response, and may be transposed in the near future for the development of extraction methods for similar analytes. The µSPEed cartridges, containing 3 µm particles, moves the solid-phase extraction (SPE) technique close to HPLC systems, both in regard to the cleanness of performances and automatability. They can be reused multiple times and coupled with the ePrep One workstation, which can eventually be directly connected to the HPLC injection port, allowing the method to be easily expanded to larger batch sizes. At the same time, the growing number of sorbent chemistries and different particle sizes might extend the application of this workflow, theoretically, to any kind of analyte in any biological fluid.

### 2.1. Method Development

**Figure 1:**
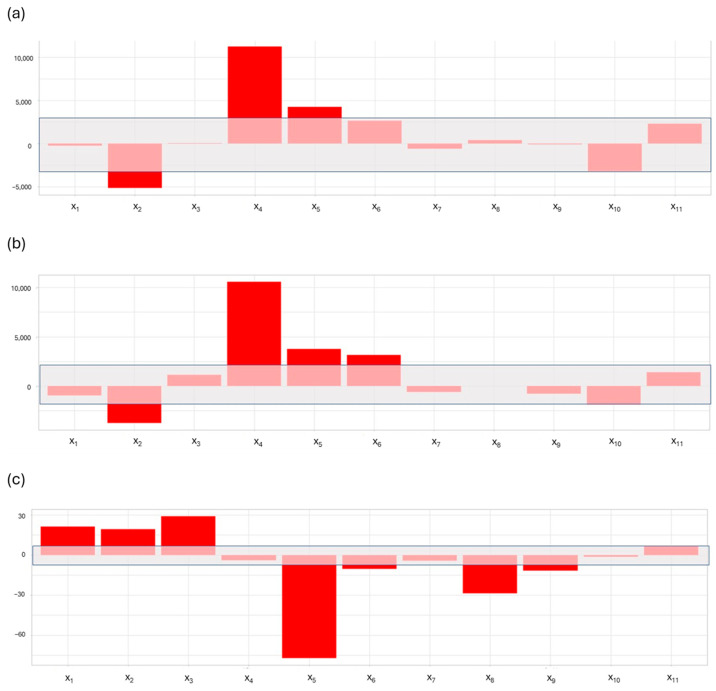
Plots of the models’ coefficients’ magnitude and sign obtained for each response. (**a**) refers to the model computed for the sensitivity of cyclophosphamide. (**b**) refers to the model describing the sensitivity of iphosphamide. (**c**) refers to the model for the duration of the sample extraction. LEGEND: x_1_, volume of methanol utilized to condition the µSPE cartridge. x_2_, volume of KOH basified water utilized to equilibrate the µSPE cartridge. x_3_, volume of the washing solution. x_4_, composition of the washing solution. x_5_, sample loading speed. x_6_, dispensing speed for the conditioning step. x_7_, dispensing speed for the equilibration step. x_8_, dispensing speed for the washing step. x_9_, dispensing speed for the elution step. Factors x_10_ and x_11_ are fictitious (dummy) factors used to estimate the random error in the experiments. The grey area in the plots shows the magnitude of the random error estimated using the coefficients of the dummy factors. So in (**a**), factors x_1_, x_3_, x_6_, x_7_, x_8_, and x_9_, having coefficients smaller than that of factor x_10_, are considered insignificant since they have effects smaller than that produced by random error. In (**b**), for the same reason, factors x_1_, x_3_, x_7_, x_8_, and x_9_, are shown as not significant since they have coefficients smaller than that of dummy factor, x_10_. In (**c**), factors x_4_ and x_7_ have coefficients smaller than that of dummy factor x_11_.

Method development was undertaken by applying a Plackett-Burman 12-run design. From the data obtained by applying a Plackett-Burman design, it was clear that the conditions needed to perform the sample extraction, allowing the highest sensitivity for both the analytes, were mainly affected by the volume of the washing solution step (x_2_), the composition of the washing solution (x_4_), and the sample loading speed on the cartridge (x_5_), as can be seen in Figure 1. The duration of the sample treatment, instead, depends mainly on the speed of loading and eluting steps and, thus, on the volumes utilized in these steps.

The multilinear regression model describing the sensitivity of the internal standard (IS) was not significant, and thus, it is not reported and only briefly mentioned here. None of the model’s coefficients were statistically significant, meaning that the selected factors (x_1_–x_9_) have no impact on the IS peak area. Therefore, slight modifications to the extraction procedure do not affect the IS peak area. This observation supports the conclusion that the method is robust as regards the isolation and detection of the IS. So, this model could be neglected and the study can be focused on the one describing the sensitivity of the analytes.

The model describing the sensitivity of cyclophosphamide has been validated (confidence interval of 95%) because the experimental response (65,490 ± 10,678 counts) was not significantly different from the predicted one (59,339 ± 10,678 counts) at the test points. In particular, the cyclophosphamide peak area was greatest when (factors are listed according to the significance of their coefficients):

- the composition of the washing solution is 90:10 of H_2_O/MeOH (the highest level for factor x_4_);
- the volume of basic water utilized to equilibrate the µSPE cartridge is 0.2 mL (the lowest level for factor x_2_);
- the sample loading speed is 25 µL/s (the highest level for factor x_5_).

The model describing the sensitivity of iphosphamide is validated (confidence interval of 95%) with an error of prediction of about 12% (experimental response of 65,490 ± 10,678 counts vs. the predicted response of 48,439 ± 10,678 counts). The coefficients’ magnitude and sign are perfectly consistent with those of the cyclophosphamide model. In this case, the factors that cause the peak area to increase are (listed in order of significance):

- the composition of the washing solution is 90:10 of H_2_O/MeOH (the highest level for factor x_4_);
- the volume of water utilized to equilibrate the µSPE cartridge is 0.2 mL (the lowest level for factor x_2_);
- the sample loading speed is 25 µL/s (the highest level for factor x_5_);
- the dispensing speed4 for the conditioning step is 55 µL/s (the highest level for factor x_6_).

The model describing the duration of the sample extraction is not validated (confidence intervals of 95% probability are: for the experimental response, 716 ± 9 s and for the predicted response, 804 ± 9 s) but the reason for this is that the experiments at the test points provided results of high precision (RSD 1.3%). So, in our opinion, although the model is not validated, the screening results provide important information that can be used to minimize the time interval for the extraction. The duration of the extraction is the shortest when:

- the sample loading speed is 25 µL/s (the highest level for factor x_5_);
- the volume of washing solution is 0.2 mL (the lowest level for factor x_3_);
- the dispensing speed of the washing solution is 25 µL/s (the highest level for factor x_8_);
- the volume of methanol utilized to condition the µSPE cartridge is 0.2 mL (the lowest level for factor x_1_);
- the volume of water utilized to equilibrate the µSPE cartridge is 0.2 mL (the lowest level for factor x_2_);
- the dispensing speed for the conditioning step is 55 µL/s (the highest level for factor x_6_);
- the dispensing speed for the eluting step is 25 µL/s (the highest level for factor x_9_).

**Figure 2:**
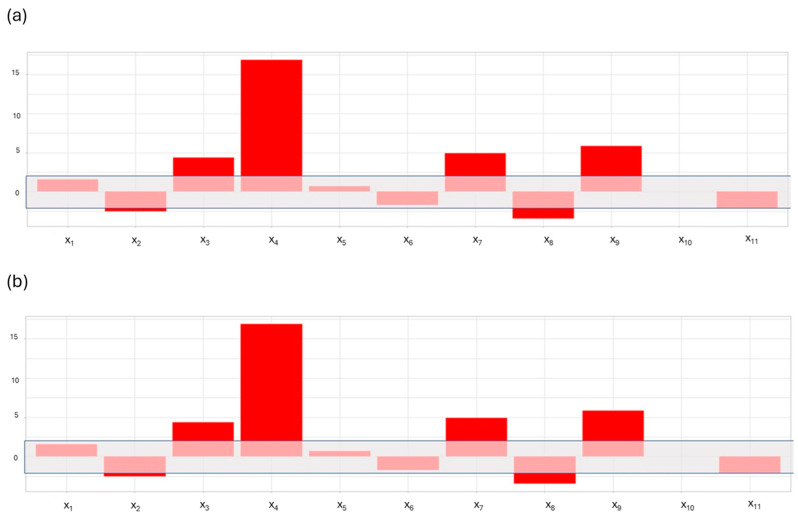
Plots of the coefficients computed for the S/N ratio models of cyclophosphamide (**a**) and iphosphamide (**b**). LEGEND: x_1_, volume of methanol utilized to condition the µSPE cartridge. x_2_, volume of basified water utilized to equilibrate the µSPE cartridge. x_3_, volume of the washing solution. x_4_, composition of the washing solution. x_5_, sample loading speed. x_6_, dispensing speed for the conditioning step. x_7_, dispensing speed for the equilibration step. x_8_, dispensing speed for the washing step. x_9_, dispensing speed for the elution step. Factors x_10_ and x_11_ are fictitious (dummy) factors used to estimate the random error in the experiments. The grey area shows the magnitude of the random error estimated using the dummy factor x_10_. Factor x_1_, having a coefficient smaller than that of factor x_10_, is considered as not significant since it has an effect smaller than that produced by random error.

Moreover, the responses for the S/N ratio of cyclophosphamide and iphosphamide were computed, and their models were validated at the confidence interval of 95%. As can be seen in Figure 2, the models are very similar and the factors that affect this ratio the most are x_3_ and x_4_, which should be set at the highest level in order to maximize the S/N ratio. The other factors have slight differences between the two models, but their contribution to the variation of the S/N ratio is very low.

In conclusion, the experimental design provides valid models to describe the sensitivity of the analytes, allowing the setting up of the sample extraction by performing a few experiments.

The experimental conditions selected were: volume of methanol utilized to condition the µSPE cartridge (x_1_) = 200 µL; volume of basified water utilized to equilibrate the µSPE cartridge (x_2_) = 200 µL; volume of the washing solution (x_3_) = 500 µL; composition of the washing solution (x_4_) = 90:10 H_2_O:MeOH; sample loading speed (x_5_) = 25 μL/s; dispensing speed for the conditioning step (x_6_) = 55 μL/s; dispensing speed for the equilibration step (x_7_) = 55 μL/s; dispensing speed for the washing step (x_8_) = 25 μL/s; dispensing speed for the elution step (x_9_) = 10 μL/s.

### 2.2. Sample Preparation and µSPE

The phenyl, polystyrene divinylbenzene (PS/DVB RP), was chosen as sorbent material from among the available ones due to its resistance to the wide range of pH values which need to be tested to maximize the extraction of the analytes. The tests carried out on the pH of extraction showed a decreasing intensity for the signal of the analytes with pH lowering, which might be explained both by the presumed basic nature of the compounds, and thus the higher percentage of the unprotonated form, and by the higher quantity of precipitate that formed during the base addition, which could lead to a higher extraction capacity of the µSPE cartridge. The tests were repeated after the method development phase, and it was confirmed that the best choice for extraction pH of the urine was 11. 

### 2.3. Performance Evaluation

**Figure 3:**
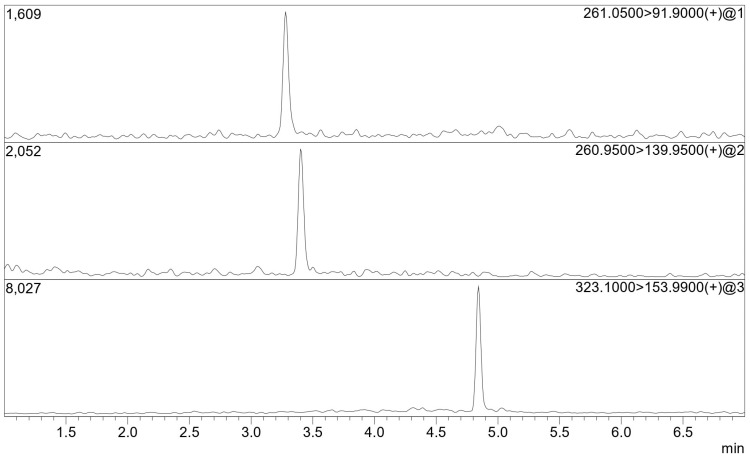
Stacked view of the experimental chromatograms of quantification ions of iphosphamide (**top**), cyclophosphamide (**centre**) and trophosphamide (**bottom**) obtained for the 10 pg/mL calibration level.

The performance evaluation results, which are summarized in Table 1, show that the required sensitivity was reached, with LOQs of approximately 9 pg/mL. This method, along with the one proposed by Izzo et al. [17], presents the lowest LOQs and solvent volumes that can be encountered in the literature for the analytes, without the need for further concentration steps on the extract. The calculated precision was comprehended between 18.7% and 21.6%, while the accuracy was between 102% and 111%. An example of the obtained experimental chromatograms is reported in Figure 3. The obtained values for matrix effect and recovery are 93% and 77% for iphosphamide and 88% and 79% for cyclophosphamide, respectively.

**Figure 4:**
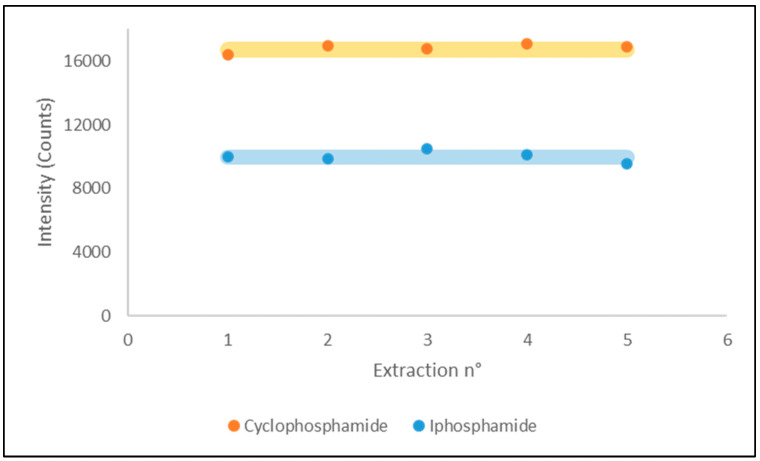
Measured intensities for both analytes for five consecutive extractions on a single µSPEed cartridge.

As can be seen in Figure 4, the tests on µSPEed showed that, in the developed conditions, a single cartridge can be reused up to five times, with a relative standard deviation lower than 5% for both analytes, before observing a reduction in their performances.

### 2.4. On-Field Method Application

Of the 23 samples assayed for the present study, two were positive for iphosphamide (27 and 182 pg/mL), while one was positive for cyclophosphamide (95 pg/mL). Even if the relatively limited number of operators monitored during the present study does not permit extensive considerations, the encountered positivities confirm the need for sensitive and high-throughput biological monitoring surveillance, which will allow further clinical investigation of Ads’ occupational hazard and provide the basis for the introduction of safe threshold values.

### 2.5. Greenness Evaluation

Nowadays, resource sustainability and environmental protection have gained great importance; thus, the evaluation of the green character of an analytical protocol must be taken into account. 

Different tools for assessing the greenness of the analytical procedure are available online [26], so an evaluation of the one which best fits the purpose of the authors has been performed. The oldest one, the National Environmental Methods Index (NEMI) [27] was discarded because it works with chemicals which are reported in official lists, such as EPA TRI list, and antiblastic drugs are not included in it. Moreover, this tool does not consider the consumption of chemical and reagent, and the amount of waste generated. Another interesting approach is the Analytical Eco-Scale [28] which is based on penalty points attributable to each step of the analytical process and then subtracted from a base of 100, but the main drawback is the inability to discriminate between the macro- and microscale of method applications. An additional interesting tool to determine the sustainability of a method is the Analytical Method Greenness Score (AMGS) calculator [29], which is not an absolute measure of the method greenness in that it considers only the environmental impact of the instrumental determination of a sample, while the sample pre-treatment is not taken into account.

**Figure 5:**
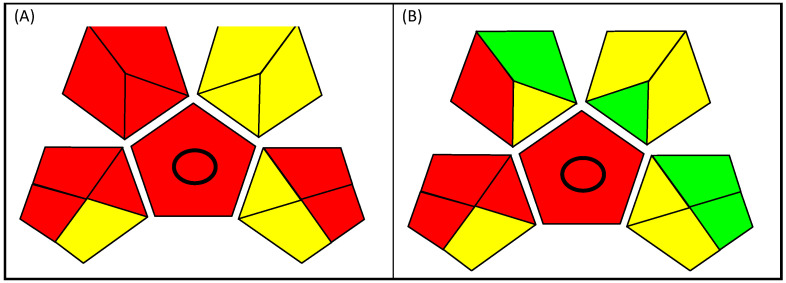
(**A**) reports the GAPI pictogram of the method in use in the authors’ laboratory, while (**B**) is one of the proposed microextraction procedures [performed with the software available at: https://mostwiedzy.pl/en/justyna-plotka-wasylka,647762-1/gapi (accessed on 27 September 2023)].

Last, being the most complete tool to the authors’ knowledge, the Green Analytical Procedure Index (GAPI) was utilized [25] to highlight that the microextraction procedure developed in this work in addition to being safer for the operators and faster in the sample preparation, also complies with the green chemistry principles. Figure 5 shows the pictograms of the two methods, and it is quite clear how the procedure involving microextraction has a greener character.

In this approach, each step of the analytical procedure is identified by a pentagram using a colour scale from red to green to indicate high or low environmental impact. As shown in Figure 5, the first pentagram at the bottom left is equal for the two methods because the sample collection and storage are the same for both procedures. While concerning the sample pre-treatment (pentagram at the upper left) and the reagent usage (pentagram at the upper right) we have the main differences, involving the microextraction approach’s less amount of sample and reagents, and less sample manipulation by the operator. The pentagram at the bottom right refers to the instrumentation step which differs only in the amount of waste generated and the hazard for the operator.

## 3. Materials and Methods

### 3.1. Chemicals

Acetonitrile, water, and methanol ultrahigh-performance liquid chromatography/mass spectrometry (ULC/MS) of purity grade were purchased from Biosolve Chimie SARL (Dieuze, France). Formic acid LC/MS grade was purchased from Carlo Erba reagents (Milan, Italy). Cyclophosphamide monohydrate, iphosphamide, ammonium formate, and potassium hydroxide 1 M solution were gradient-grade HPLC reagents or better, purchased from Merck KgaA (Darmstadt, Germany). Trophosphamide (purity > 95%), used as internal standard (IS), was purchased from CliniSciences (Guidonia Montecelio (RM), Italy).

### 3.2. Instruments

The LC system consists of a Shimadzu Nexera X2, equipped with a DGU-20A5R degasser unit, two LC-30AD pumps, SIL-30AC autosampler, CBM-20A system controller, and CTO-20AC column oven, coupled through ESI with a Shimadzu LCMS 8050 triple quadrupole (Shimadzu Corp., Kyoto, Japan).

The software, LabSolution^®^ ver. 5.97 (Shimadzu Corp., Kyoto, Japan), was used to perform instrument control and data acquisition.

Sample extraction was performed using an ePrep^®^ ONE workstation, equipped with a 500 µL ePrep eZy-Connect (µSPEed^®^) Syringe (P.N. 01-09083) and PS/DVB RP, 3 µm/300Å µSPEed^®^ Cartridges (P.N. 01-10151). The software used to operate the workstation was ePrep AXIS Software (ver. 1.24.19).

### 3.3. Chromatographic Conditions and Mass Spectrometry Parameters

The UHPLC mobile phase consisted of 4mM ammonium formate 0.021% formic acid water solution (A) and an acetonitrile:methanol 90:10 (*v*/*v*) mixture with 0.021% formic acid addition (B). The elution was carried out at a constant flow rate of 0.55 mL/min, applying a 7 min linear gradient from 10 to 85% of B.

The chromatographic column was a Cortecs^®^ UPLC T3, 500 × 2.1 mm (Waters Corporation, Milford, MA, USA), packed with material made of core-shell particles of 1.6 μm diameter. The total analysis time was 13.2 min.

The settings of the ESI source, operating in positive ion mode, were the following: interface voltage 4 kV, nebulizing gas flow 3 L/min, heating gas flow 10 L/min, interface temperature 400 °C, desolvation temperature 650 °C, desolvation line temperature 300 °C, heat block temperature 500 °C, and drying gas flow 10 L/min. The tandem mass spectrometry acquisition was set to multiple reaction monitoring (MRM) with a dwell time of 63 msec. The following fragmentations were selected as quantifier and reference transitions, respectively: for cyclophosphamide 260.95 > 139.95 [−22 V], 260.95 > 105.9 [−21 V]; for iphosphamide 261.05 > 91.9 [−23 V], 261.05 > 153.9 [−22 V]; for trophosphamide 323.1 > 153.99 [−24 V], 323.1 > 106.1 [−21 V].

### 3.4. Standard Solutions and Calibration Levels

Stock solutions of iphosphamide, cyclophosphamide, and trophosphamide were prepared at 1 mg/mL using a mixture of H_2_O/MeOH 50:50 (*v*/*v*) and were stored at −20 °C. A mix solution containing 1 µg/mL of iphosphamide and cyclophosphamide was prepared in H_2_O from the stock solutions, then diluted with water at 5 ng/mL to obtain the STD work solution. A trophosphamide solution was prepared at 1 µg/mL in H_2_O from the stock solutions, then diluted with water to 15 ng/mL to obtain the IS work solution.

Starting from stock solutions, a working solution containing 5 ng/mL of iphosphamide and cyclophosphamide in H_2_O and a 15 ng/mL of SI working solution in H_2_O were prepared. 

A six-level calibration curve was prepared by adding 5 µL of IS work solution, the appropriate volume of STD work solution, and a blank urine pool to reach a final volume of 1.5 mL. The analyte concentrations in the calibration solutions were: 0, 5, 10, 20, 35, and 50 pg/mL. An internal quality control solution (CQI) was prepared at 15 pg/mL of analytes and 50 pg/mL of IS.

### 3.5. Sample Preparation

The samples analyzed for method development were prepared using the following procedure: a 300 mL blank urine pool was collected from workers who were not exposed to antineoplastic drugs; it was thus divided into two portions and spiked with analytes and IS solutions to obtain a 200 mL part containing 100 pg/mL of cyclophosphamide, iphosphamide, IS, and a 100 mL part containing only 100 pg/mL of IS; the two portions were divided in 5 mL aliquots, stored at −80 °C, and analyzed through LC-MS/MS according to the DoE experimental plan.

Due to a lack of uniformity for the analytes’ pka values, which can be found in the literature [30,31,32,33] or predicted with different software programs [34,35], the pH of extraction was tested before and after the method setup by extracting spiked urine samples at three different pH values, respectively 3, 7 and 11, and monitoring the presence of the analytes in all the steps of the extraction. 

A 1.5 mL volume of urine was collected in a 5 mL polypropylene tube and added to 150 µL of a 1M KOH solution and 5 µL of a 15 pg/mL IS solution, then centrifuged at 800× *g* for 10 min and filtered through a 0.2 µm GHP Acrodisc^®^ syringe filter (Pall Corporation, Long Island, NY, USA) before the extraction procedure. The same procedure was also applied to pooled urine used to prepare calibration levels.

The extraction method was also tested on 23 operators involved in AD handling from four Italian hospitals by retrieving 24 h urine samples. Aliquots from the samples were collected and analyzed through the developed method.

### 3.6. Chemometric Evaluation

The application of a Plackett-Burman design allowed us to evaluate the effect of 9 factors on sample extraction by performing 12 experiments. 

The responses selected to develop the method were four in number: the peak areas of the analytes (cyclophosphamide, iphosphamide, and internal standard) and the duration of the sample extraction. The aim of the experimental design was to maximize the peak areas, both for the analytes and the internal standard, which means maximizing their sensitivity and, at the same time, minimizing the sample extraction duration.

The factors evaluated to study the sample extraction on the ePrep ONE workstation were the volume of methanol utilized to condition the µSPE cartridge (x_1_), the volume of basic water utilized to equilibrate the µSPE cartridge (x_2_), the volume of the washing solution (x_3_), the composition of the washing solution (x_4_), the sample loading speed (x_5_), the dispensing speed for the conditioning step (x_6_), the dispensing speed for the equilibration step (x_7_), the dispensing speed for the washing step (x_8_), and the dispensing speed for the elution step (x_9_). Two dummy factors were added to the experimental plan (Table 2, factors x_10_ and x_11_) to estimate the random error; additionally, three validation points (experiments 1, 6, and 7) were randomly chosen to assess the concordance between the experimental observations and the model predictions.

The data were collected using Microsoft Excel and processed using Rstudio Version 1.2.1335 ©2009–2019 Rstudio, Inc., as GUI for R version 3.6.1 (5 July 2019) “Action of the Toes”, ©2019 (Rstudio PBC, Boston, MA, USA). The R Foundation for Statistical Computing.

### 3.7. Performance Evaluation

To evaluate the performances of the method, three sets of calibration samples and CQI were freshly prepared and analyzed every day for six days to control the interday precision, whereas for the intraday precision, six sets were prepared and analyzed in a single day. Calibration curves were obtained by plotting the peak area ratios (PAR) between the analyte and IS quantitation ions versus the nominal concentration of the calibration solution. A linear regression analysis was applied to obtain the best-fitting calibration curve. The limits of detection and quantitation (LOD and LOQ) were calculated according to ICH guidelines using the approach based on the standard deviation of the intercept and slope of the regression [36].

The method precision was evaluated through the relative standard deviation (RSD%) of the replicate analysis of CQIs. The accuracy was determined by the recovery ratio percentage (RE%) computed between the determined and theoretical amounts of the replicate analysis of the CQIs.

Three sets of four replicate samples were prepared to evaluate each analyte’s matrix effect (ME) and RE%. Set1 was prepared by adding 30 µL of STD work solution to a 1.5 mL blank urine sample to obtain a concentration of 100 pg/mL and then proceeding to extraction; Set2 was prepared by extracting blank pool urine samples and adding 5 µL of ME mix solution to 195 µL of extract; Set3 was prepared by diluting 5 µL of ME mix solution with 0.1% formic acid MeOH:H_2_O 90:10 solution up to a concentration of 500 pg/mL [37].

ME and RE% figures were calculated for each analyte by comparing the mean results of Set1, Set2, and Set3 according to the formula:

(1) $$ME\left(\%\right)=\frac{Set2}{Set3}\times 100$$

(2) $$RE\left(\%\right)=\frac{Set1}{Set2}\times 100$$

### 3.8. µSPE

The solutions used for µSPE steps were: 250 mL of a KOH 0.01 M, 250 mL of a 1% formic acid MeOH:H_2_O 90:10, 100 mL of a KOH 0.01 M H_2_O:MeOH 90:10, and 100 mL of a KOH 0.01 M H_2_O:MeOH 95:5.

The extraction method consisted of the following steps: 200 µL methanol conditioning (dispense velocity 55 μL/s), 200 µL of KOH 0.01 M equilibration (dispense velocity 55 μL/s), two times 500 µL of basified urine sample (sample loading velocity 25 μL/s) for a total of 1 mL of urine loaded, 500 µL of KOH 0.01 M H_2_O:MeOH 90:10 wash (dispense velocity 25 μL/s), 200 µL of 1% formic acid MeOH:H_2_O 90:10 elution (dispense velocity 10 μL/s), and 300 µL of methanol wash (dispense velocity 15 μL/s). All the aspiration flow rates were set at 15 μL/s. After vortex mixing, 5 µL of the eluted solution was injected for the UHPLC-MS/MS analysis. The extraction time for each sample was 9.5 min.

**Figure 6:**
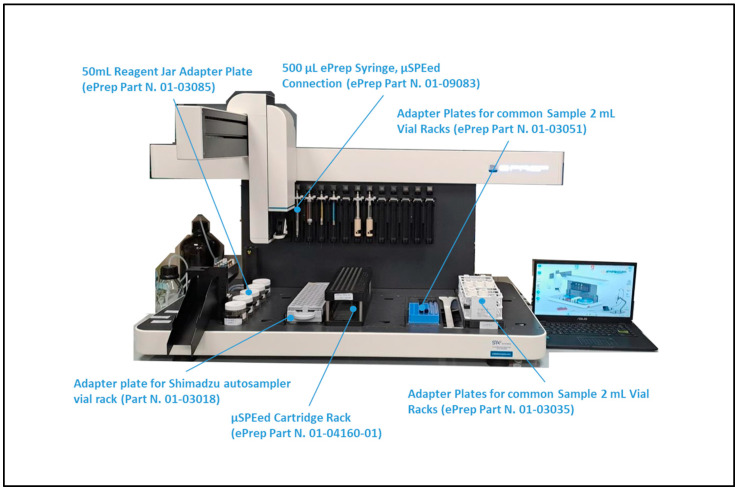
Configuration used for the ePrep ONE workstation.

The ePrep ONE workstation was equipped with a 2 mL glass vial sample rack (ePrep Part N. 01-03035), Shimadzu autosampler rack adapter plate (ePrep Part N. 01-03018), 50 mL Reagent Jar Adapter Plate (ePrep Part N. 01-03085), μSPEed Cartridge Rack (ePrep Part N. 01-04160-01), Adapter Plates for common Sample 2 mL Vial Racks (ePrep Part N. 01-03051), 500 μL ePrep Syringe, and μSPEed Connection (ePrep Part N. 01-09083), as shown in Figure 6.

### 3.9. Greenness Evaluation

The environmental impact of the analytical method was evaluated by comparing it to the procedure currently in use inside the research laboratory, which involves the use of conventional SPE cartridges [38]. The comparison was performed following the instruction of the Green Analytical Procedure Index (GAPI) tool. All the analytical steps except the sample preparation were held constant to focus the results on the extraction procedure.

## 4. Conclusions

The healthcare personnel of compounding and administration units are nowadays daily exposed to hazardous medicinal products such as Ads, and the use of these drugs is progressively expanding. Biological monitoring is a strong tool for occupational health, but increasingly sensitive methods are necessary to keep up with the low concentrations which can be encountered in urine samples. Over the years, many methods for the determination of the most common ADs (such as cyclophosphamide and iphosphamide) have been proposed, and yet most of them require great amounts of samples and solvents to reach the sensitivity of pg/mL, along with multiple sample preparation and concentration steps.

We here propose a µSPE automated method that, coupled with UHPLC-MS/MS analysis, can determine urine contaminations of cyclophosphamide and iphosphamide in the order of 10 pg/mL, with an automated extraction time of less than 10 min. The chemometric development strategy, which allows the reduction in the number of experiments needed for the setup to 12, might be used as a scaffold for future applications of ePrep µSPEed cartridges in the expansion of monitored substances.

The developed method shows adequate accuracy and precision in the range of the LOQ. Furthermore, the need for low volumes of solvents and possibility to use a single cartridge up to five times, makes the µSPEed extraction approach in line with the current and future developments of green chemistry.

The analytical procedure was successfully applied to urine samples from compounding wards of four Italian hospitals and could be implemented on a large scale to allow further clinical investigation of Ads’ occupational hazard and provide the basis for the introduction of safe threshold values.

## Figures and Tables

**Table 1:** Performance evaluation results, expressed as the limit of detection (LOD), the limit of quantification (LOQ), precision (PR) and accuracy (ACC) of the method, extraction recovery (RE), and matrix effect (ME).

Compound	Slope	Intercept	R^2^	LOD (pg/mL)	LOQ (pg/mL)	PR (%)	ACC (%)	RE (%)	ME (%)
Iphosphamide	0.019	0.055	0.996	2.87	8.6	18.7	102	77	93
Cyclophosphamide	0.020	0.094	0.995	3.12	9.4	21.6	111	79	88

**Table 2:** Experimental plan applied in the study. The experiments are listed in the random order applied during the working session.

#	x_1_ (mL)	x_2_ (mL)	x_3_ (mL)	x_4_ (%MeOH, *v*/*v*)	x_5_ (µL/s)	x_6_ (µL/s)	x_7_ (µL/s)	x_8_ (µL/s)	x_9_ (µL/s)	x_10_ (Dummy)	x_11_ (Dummy)
1	0.35	0.35	0.35	5	15	55	55	15	15	0	0
2	0.5	0.2	0.2	0	25	15	55	25	5	1	1
3	0.5	0.2	0.5	10	5	55	55	25	5	−1	−1
4	0.2	0.2	0.5	0	25	55	15	25	25	1	−1
5	0.2	0.5	0.2	10	25	15	55	25	25	−1	−1
6	0.35	0.35	0.35	5	15	55	55	15	15	0	0
7	0.35	0.35	0.35	5	15	55	55	15	15	0	0
8	0.5	0.5	0.2	10	25	55	15	5	5	1	−1
9	0.2	0.2	0.2	0	5	15	15	5	5	−1	−1
10	0.2	0.2	0.2	10	5	55	55	5	25	1	1
11	0.5	0.2	0.5	10	25	15	15	5	25	−1	1
12	0.2	0.5	0.5	0	25	55	55	5	5	−1	1
13	0.2	0.5	0.5	10	5	15	15	25	5	1	1
14	0.5	0.5	0.2	0	5	55	15	25	25	−1	1
15	0.5	0.5	0.5	0	5	15	55	5	25	1	−1

LEGEND: #, experiment number. x_1_, volume of methanol utilized to condition the µSPE cartridge. x_2_, volume of basic water utilized to equilibrate the µSPE cartridge. x_3_, volume of the washing solution. x_4_, composition of the washing solution. x_5_, sample loading speed. x_6_, dispensing speed for the conditioning step. x_7_, dispensing speed for the equilibration step. x_8_, dispensing speed for the washing step. x_9_, dispensing speed for the elution step. x_10_, x_11_ dummy factors.

## Data Availability

Data are contained within the article.
